# *Nigella sativa* and Its Active Compound, Thymoquinone, Accelerate Wound Healing in an In Vivo Animal Model: A Comprehensive Review

**DOI:** 10.3390/ijerph17114160

**Published:** 2020-06-11

**Authors:** Nusaibah Sallehuddin, Abid Nordin, Ruszymah Bt Hj Idrus, Mh Busra Fauzi

**Affiliations:** 1 Tissue Engineering Centre, Faculty of Medicine, Universiti Kebangsaan Malaysia, Cheras, Kuala Lumpur 56000, Malaysia; nus_90@hotmail.my (N.S.); ruszyidrus@gmail.com (R.B.H.I.); 2 Department of Physiology, Faculty of Medicine, Universiti Kebangsaan Malaysia, Cheras, Kuala Lumpur 56000, Malaysia; m.abid.nordin@gmail.com

**Keywords:** *Nigella sativa*, thymoquinone, skin wound healing, anti-inflammatory, antioxidant, antibacterial

## Abstract

*Nigella sativa* (NS) has been reported to have a therapeutic effect towards skin wound healing via its anti-inflammatory, tissue growth stimulation, and antioxidative properties. This review examines all the available studies on the association of *Nigella sativa* (NS) and skin wound healing. The search was performed in Medline via EBSCOhost and Scopus databases to retrieve the related papers released between 1970 and March 2020. The principal inclusion criteria were original article issued in English that stated wound healing criteria of in vivo skin model with topically applied NS. The search discovered 10 related articles that fulfilled the required inclusion criteria. Studies included comprise different types of wounds, namely excisional, burn, and diabetic wounds. Seven studies unravelled positive results associated with NS on skin wound healing. Thymoquinone has anti-inflammatory, antioxidant, and antibacterial properties, which mainly contributed to wound healing process.

## 1. Introduction

### 1.1. Burden of Wound Healing

Skin wound healing has long been the focus of regenerative medicine, partly due to the accessibility of the skin tissue and its inert ability to regenerate [1]. With the treatment costs ranging from 28.1 billion dollars to 96.8 billion dollars for 8.2 million Medicare beneficiaries, innovation in the wound healing technology is paramount [2]. In recent years, the burden of wound healing management has increased with the emergence of antibiotic-resistant bacteria that can impede the wound healing process [3]. Consequently, the use of a natural product such as *Nigella sativa* (NS) in the management of wound healing has been proposed. 

### 1.2. Therapies for Skin Wound Healing

Even though damaged skin has self-regeneration capability as a native mechanism automatically activated in the human body, the presence of specific unavoidable conditions such as chronic wounds, burn wounds, or non-healing ulcers could hinder the healing process [4]. Thus, the interruption of the normal healing phase could lead to other phases of a chronic state that indirectly increase the high susceptibility to infection and finally affect the patient’s quality of life. 

Briefly, the available wound care therapies can be categorized into modern-based and traditional-based treatment. However, the combination of both approaches could lead to better outcomes in wound care management that are currently being extensively investigated worldwide. The traditional-based treatment, known as alternative or complimentary medicine, usually involves plant-/herbal-derived compounds, animal-derived products, living organisms, and, lastly, silver dressing. The venture of this traditional-based medicine specifically on plant-based products is currently found to be a highly potent alternative for the skin treatment to cater to some limitations under modern treatment. 

The limitations include the longer manufacturing period, high cost, and susceptibility to bacterial resistance. Thus, the availability of this traditional-based treatment in current wound management provides a balanced condition for the acceleration of the healing process with the presence of healing characteristics such as anti-inflammatory, antioxidant, and etc. However, this treatment could not permanently replace the tremendous use of current effective drugs and cellular therapies for instance, which then calls for the combination use of traditional medicine and modern products. 

It has been proved previously that the combination treatment of absorbable oxidized regenerated cellulose with NS expedites wound healing in a diabetic model and promotes less scaring [5]. Moreover, a modern-based treatment by using laser was demonstrated to be effective on the inflammatory and proliferative phase with the presence of fibroblasts and the appearance of high collagen density compared to *Nigella sativa* [6]. Therefore, to this point, the plant-based approach together with modern therapy could amplify the synergistic effects towards the wound healing process.

### 1.3. Nigella sativa and Its Bioactive Component in Skin Wound Healing

*Nigella sativa* (NS) has had a special place as a medicinal herb since the ancient time. This is mostly because in Islamic-based medicine, NS has been mentioned by Prophet Muhammad PBUH as the cure for all diseases except death [7]. Furthermore, it is also mentioned in the Bible and other religious books as well, particularly with regard to its therapeutic effect [8].

NS is referred to as black cumin or black seed, in English and as “Habbatussauda”, in Arabic. It can be found in Southern Europe, North Africa [9], and Asia including Syria, Turkey, Saudi Arabia, Pakistan, and India [10]. The fruit of NS is a capsule-based fruit containing numerous black trigonal seeds [11].

NS and thymoquinone (TQ) are well known to have a wide range of therapeutic effects mainly attributed to their anti-inflammatory, antioxidant, anticancer, antibacterial, nephroprotective, and neuroprotective properties that have been revealed in various in vivo studies [12,13].

The positive effects of NS in skin wound healing is mainly due to the induction of angiogenesis, increased fibroblast proliferation, and subsequent collagen synthesis [14]. Moreover, NS has been reported to reduce the presence of white blood cells, tissue damage, and bacterial infection [15]. 

The most prominent active component of NS is thymoquinone (TQ) [16]. According to a review performed by Khader et al., there are four favourable properties that describe TQ based on the 406 empirical evidences evaluated. These properties are anti-inflammatory, antioxidant, anticancer, and hepatoprotective [17]. In addition, Darakhshan et al. stated that TQ possess antihistaminic, antimicrobial, gastroprotective, nephroprotective, and neuroprotective properties [18].

### 1.4. Wound Healing Cascade

The wound healing process involves four consecutives, yet overlapping, phases comprising haemostasis, inflammatory, proliferative, and remodelling phases [19]. Any disruption or imbalance in each phase can result in wound healing attenuation or over induction. Excessive inflammation found in diseases such as type 2 diabetes or peripheral vascular diseases can lead to attenuation of the process and result in delayed wound healing [20]. Alternatively, an excessive proliferation can lead to the over induction of healing and result in scar or keloid formation.

### 1.5. Wound Healing Models

In order to evaluate the efficacy of a wound healing therapy, many wound healing models have been developed. At the pre-clinical level, animal models for various skin depth and co-morbidity were available [21]. In terms of wound depth, a wound can be either a superficial, partial-thickness, or full-thickness wound [22]. Superficial wounds occur only at the epidermis level, such as abrasion, while partial-thickness wounds involve the epidermis and dermis, such as lacerations or burn wounds. Lastly, full-thickness skin wounds involve all skin layers and may extend to muscles and bones, such as incision wounds or gunshot wounds. 

Regarding co-morbidity, there are many conditions that contribute to the worsening of the wound healing impairment [23]. These include diabetes mellitus, immunocompromised disease, severe bacterial infection, and others. Therefore, under these particular conditions, further clinical interventions are required to reduce their symptoms and complications. Any failure in wound healing within three months of skin injury is considered a chronic wound [24]. Alternatively, acute wounds are rapid injuries that happened suddenly due to trauma including abrasion, laceration, penetrating, degloving, or burn injuries [25].

### 1.6. Wound Healing Parameters

Therapeutic outcomes of wound healing intervention can be evaluated from the combination of macroscopic and histological observations, biochemical and biomechanical measurements, as well as the evaluation of cellular and immunologic responses to estimate the effect of a therapy on the progression of wound repair [26].

The macroscopic evaluation, also known as gross morphology observation, provides an early indication of wound healing efficiency. Parameters that can be obtained from this observation include time to complete healing, wound contraction, and wound closure [27]. It is important to note that wound closure does not guarantee quality skin regeneration [28]. As such, a microscopic evaluation of the skin regeneration is paramount. 

A major advantage of using animal models of wound healing is the ability to harvest the skin tissue for histological observation [21]. Briefly, a portion of skin (various sizes depending on the wound area created initially) is excised from the euthanized animal model and fixed with 10% formalin prior to serial tissue processing, followed by a paraffin blocking process [29]. The fixative used will maintain the structure of the harvested skin before further staining with a specific stain such as Masson trichrome and haematoxylin and eosin. The selection of the fixative for different analyses is crucial to ensure that the integrity and microstructure have been preserved to avoid any biased data. 

The various animal models such as bovine, sheep dog, porcine, zebrafish, etc. have been selected to evaluate the therapeutic effect of any treatment for wound repair in different condition such as diabetic condition, metabolic syndrome, and burn condition with infection. Grada et al. (2018) described the various in vivo models commonly used to assess the efficiency of treatment for wound healing, primarily depending on the merits and limitations of each model according to the experimental objectives [30]. Each animal model has its own advantages and disadvantages that could make it difficult to cater to all requirements and needs for a certain evaluation. For instance, the use of a mouse model can provide a rough idea of the effectiveness on wound treatment but not reflect overall output representing the human model, as this model is dominated by myofibroblast-mediated contraction and is fundamentally different from the human skin structure [31].

Through histological observation, changes in the skin structure that represent the different phases of wound healing can be evaluated. The haemostasis phase, which starts immediately or up to approximately three hours following tissue injury to stop the bleeding, can be observed via the formation of a scab at the site of injury [32]. Next, the inflammation phase can commonly be characterized by the infiltration of immune cells, swelling, and redness within the dermis layer [33]. The inflammation phase can be acute or chronic, depending on whether it lasts a few days or months. Consecutively, the success of the proliferative phase, which may last from four hours to 14 days, can be measured through the formation of granulation tissue and epithelialization [34]. Finally, restoration of the skin tissue to resemble its pre-injury state can occur through the remodelling phase. This phase usually starts from the day eight and may last for up to one year depending on the patient’s condition and body metabolism [35].

Recent findings have associated oxidative stress with wound healing impairment [36]. In the normal condition, the production of free radicals through cell metabolism is balanced by the antioxidant enzymes such as superoxide dismutase (SOD), catalase (CAT), glutathione peroxidase (GPx), glutathione reductase (GRx), and glutathione-S-transferase (GST) [37]. Excess of this free radical can damage molecular structures such as proteins, lipids, and DNA [38]. These damages in turn result in the disruption of the normal wound healing process, which is heavily dependent on a plethora of cellular and molecular mediators. Hence, interventions that can curb these damages will have a positive effect on wound healing. 

In this review, a systematic search of the electronic databases, namely Medline via EBSCOhost and Scopus, was conducted to identify published research articles regarding the positive effect of NS or TQ towards skin wound healing. The findings were critically appraised and presented in terms of the wound healing outcome measures.

## 2. Methods

### 2.1. Literature Review

Relevant studies reporting topical effect of NS on skin wound were systematically obtained via an extensive search on the biomedical science-related databases, namely Medline via EBSCOhost (published between 1970 to March 2020) and Scopus (published between 1970 to March 2020). The search strategy was adapted from a previous publication by Nordin et al. with slight modification [39]. The search approach included a combination of the following two sets of keywords (1) *Nigella sativa* OR black seed OR black seed oil OR thymoquinone AND (2) skin OR wound healing.

### 2.2. Selection of Research Articles

Records obtained from the keyword search were filtered under three different phases by three independent reviewers before the content was evaluated according to the inclusion and exclusion criteria of this review. First, the records were limited to primary literature with abstracts written in English language. In the second phase, articles that falls within the category of secondary literature were excluded from the selection process. Finally, duplicate records were excluded.

### 2.3. Inclusion and Exclusion Criteria

For this review, only article that reported the topical effect of NS product on wound healing in an in vivo skin model were included. Articles must report the effects of at least one of these which were (1) wound size OR (2) gross appearance of wound area OR (3) histological analysis. Wound healing can occur in various skin conditions such as skin cancer, skin fibrosis, and embryonic development. These factors may hinder the role of NS in wound healing. Therefore, for this systematic review, papers that reports the effect of NS on wound healing of (1) skin cancer, (2) skin fibrosis, or (3) embryonic development were excluded from this review. 

### 2.4. Data Extraction and Management

Following record screening, the titles of the articles were examined to exclude articles that were not relevant to the proposed inclusion criteria. It was followed by the abstract’s evaluation prior to data extraction from a full paper read. Last but not least, the rest of papers were read carefully line by line, to exclude any articles that did not meet the inclusion criteria. These articles were read thoroughly by three independent reviewers, and the data collection standardization was made through the data extraction form (DEF). However, all the selected articles were agreed on by all reviewers to ensure their clarity and unbiasedness before the data extraction phase began. The details of DEF included the following information: (1) experimental model used; (2) form of NS; (3) summary of methods used; (4) summary of results; and (5) final conclusions.

## 3. Results

### 3.1. Search Results

**Figure 1:**
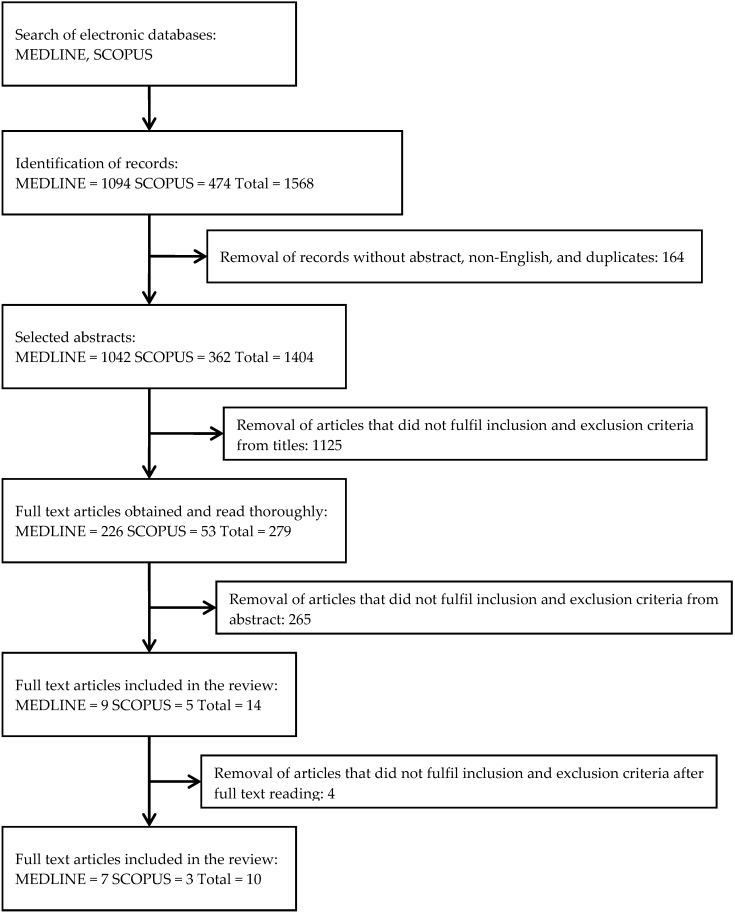
Flowchart of the selection process.

The extensive literature search successfully identified 1568 potentially relevant records. Initial screening of the records resulted in the removal of 164 records that were not original articles, not published in English language, and were duplications. The articles were then screened based on their titles for any inclusion criteria, which resulted in the removal of 1125 articles. From the remaining 279 articles, 265 articles were removed after screening the abstracts for inclusion and exclusion criteria. Reviewers then read the full text of the remaining 14 papers, of which 4 articles were excluded because they did not fulfil the inclusion criteria. Ten articles were finalized for data extraction. A flow chart of the article retrieval process is shown in Figure 1.

### 3.2. Study Characteristics

A summary of the study characteristics is shown in Table 1 and Table 2. Briefly, ten articles were included in the review. All articles were published between the years 2010 and 2020. There were four types of wound investigated among the studies, namely excision wound [14,40,41,42], diabetic ulcers [43,44,45], deep second-degree burn [46,47], and chemical burn [48].

According to wound depth, three types of wound depths were created, which were superficial partial-thickness wound [40], deep partial-thickness wound [47,48], and full-thickness wound [43,45,46]. The remaining articles [14,41,42,44] did not specify any details about the depth of wound in their studies.

Three different forms of NS were used among the studies including oil, cream, and gel. Three studies used NS oil extract in which NS seeds were cold pressed to produce oil in one study [40], while the remaining two studies did not explain the process [14,48]. Four studies demonstrated NS oil cream [41,42,45,47] as their topical approach and one study demonstrated NS oil gel [43]. To fabricate NS oil cream, three studies used fixed oil [42,45,47] while the other one did not have the information [37]. TQ was used in the two remaining studies in two forms; 10% in petroleum jelly (w/v) [44] and 0.5% solution, prepared by dissolving 500 mg TQ in 100 mL dimethyl sulphoxide (DMSO) [46].

According to geographical origin, two studies purchased their NS oil from Iran [40,45], three studies from Turkey [41,42,47], and another study from Indonesia [43] and India [14] each. Following that, one study obtained NS oil extracted by Aljouf University, Saudi Arabia [48]. Two studies were described using TQ powder as their treatment and purchased it from Sigma-Aldrich [44,46]. 

### 3.3. NS in Skin Wound Healing

#### 3.3.1. Gross Appearances

Macroscopic observation of wound area reduction acts as an early predictor of the wound healing outcome. In humans, more than 53% wound reduction in 4 weeks duration can firmly predicted the completion of wound healing in 12 weeks [49]. Accordingly, any wound that fails to reduce by half over 4 weeks of treatment is most likely complicated and considered to be a chronic wound [50]. 

Moreover, information regarding the progression through every phase of wound healing can also be obtained via gross morphology observation [51]. For instance, the inflammatory phase can be characterized by the signs of erythema, heat, oedema, pain, and functional disturbance experienced by the animal. In the proliferative phase, the development of healthy granulation tissue and re-epithelialization is commonly identified with the presence of a wound scab. As wounds start to be mature, a whitish scar tissue appears to replace the previously injured tissue [52]. Most of the studies (8 out of 10) included in this review included the gross morphology outcome.

The current gold standard for topical burn wound treatment is silver sulphadiazine (SS) that is currently commercially available [47]. To study the efficacy, Selçuk et al. compared the effect between SS and TQ in a burn wound model. In their study, TQ was applied topically, administered intraperitoneally, or in combination of both. In intraperitoneally administered TQ, the wound had a similar closure rate with SS. On the other hand, topically applied TQ with or without intraperitoneal administration exhibited superior wound healing compared to the gold standard [46].

Phonophoresis is the use of therapeutic ultrasound to facilitate the transdermal absorption of topical products such as analgesic or anti-inflammatory creams [53]. In another study utilizing a chemical burn wound model, the effectiveness of different modes of ultrasound or phonophoresis with NS oil on wound healing was investigated [48]. The treatments were compared to the moist-exposed burn ointment (MEBO), a traditional Chinese herb formulation used to alleviate pain and improve tissue regeneration in burn wounds. The study reported a significant reduction in wound size by all treatment groups, with the pulsating phonophoresis of NS oil group having the smallest wound area. Taken together, both NS and TQ exhibited a positive effect in improving wound healing outcome in burn wounds [46,48].

In terms of excisional wound, three studies reported the gross morphology outcome [40,41,42]. In the first study by Han et al., NS oil cream was compared to the *Hypericum perforatum* (HP) oil cream [41]. HP or its common name St. John’s Wort, is another traditional herb known for its anti-inflammatory properties [54]. By the end of the study duration, the NS group demonstrated a significantly smaller wound area and higher wound contraction compared to the control group but the difference was not significant compared to the HP group [41]. 

Effect of NS in excisional wounds continued to be explored by Javadi et al. in 2018 [40]. In their study, NS alone or in combination with honey was compared against phenytoin, an anticonvulsant drug that is known to enhance wound healing [55]. By measuring the necrotic tissue area 20 days post-wounding, the combination of NS and honey group exhibited the smallest necrotic tissue area followed by phenytoin, honey alone, and NS alone groups [40].

Finally, in 2019, Kumandaş et al. published the latest report on the efficacy of NS oil cream in excisional wounds. In their study, NS oil cream was compared with a zinc–silver cream. Materials with silver ions have the ability to disrupt bacterial cell wall structure, effectively preventing bacterial colonization at the wound site [56]. However, Kumandaş et al. reported slower healing rate in both NS oil and zinc–silver cream, with NS oil cream having the slowest healing rate [42]. 

In chronic delayed wound, efficacy of NS or TQ were investigated with either alloxan-induced or streptozotocin-induced diabetic rats. In 2017, Yusmin and Ahmad investigated the efficacy of TQ in petroleum jelly in an alloxan-induced diabetic wound. Through the gross morphology observation, wound contractions in TQ group was significantly greater than that in the control group at day 3 but significantly lower than the control group at day 7 and 14 [44]. This suggest that the effect of TQ was on the early phases of the wound healing. 

In the 2018 study by Sari et al., the effect of NS oil gel on alloxan-induced diabetic wound was explored. NS oil gel was compared against *Aloe vera* (AV) oil gel with no gel applied in the control group. Although AV gel demonstrated significant reduction of the wound size after 7 days when compared to no treatment, negligible effect on wound size was observed in the NS oil gel group [43]. This is contradictory to the recent study by Nourbar et al., who reported the fastest wound healing rate with NS extract treatment in their streptozotocin-induced diabetic rat on all days of treatment [45]. The enhancement of wound healing was even greater than that of the phenytoin treatment. The contrasting results may be due to different mechanism of beta cell death in streptozotocin- and alloxan-induced diabetic rats. Further studies are certainly needed to study the effects [57].

Overall, as the macroscopic progression in both NS- and its active compound (TQ)-treated group was faster than that in control group, it could be safe to conclude that both treatments accelerate wound healing progression in burn wounds and excisional wounds. However, the effect was not as robust in diabetic wounds.

#### 3.3.2. Microscopic Findings

The microscopic evaluation of the wound is important in clinical practice for better wound management. Microscopic evaluation of the wound helps to understand the effect of the wound intervention on the essential components of the healing process such as angiogenesis, inflammation, fibroplasia, granulation tissue formation, epithelialization, and differentiation [58]. Similar to the gross morphology outcome, most of the studies (8 out of 10) included in this review included the histological analysis of the regenerated tissue.

Two of the earliest studies included in this review, reported only histological assessment outcome. In terms of burn wounds, Yaman et al. reported that the wound treated with NS oil showed better anti-inflammatory cell response, granulation tissue formation, vascularization, and epithelialization compared to those of SS and control [47]. Another study that investigated the effect of NS on excisional wounds, revealed a positive effect of NS in their rabbit model. Compared to 1% pyodine antiseptic treatment, NS demonstrated greater angiogenesis, fibroblast proliferation, and collagen synthesis in the regenerated tissue [14].

The positive effects of TQ in burn wounds, observed in the macroscopic observation as reported by Selçuk et al., was supported by the microscopic findings [46]. Wounds treated with TQ showed better anti-inflammatory cell response, granulation tissue formation, vascularization, and epithelialization. Together with the Yaman et al. study, NS and TQ demonstrated better a therapeutic effect on wound healing in burn wounds [46,47].

The greater wound closure in the NS group as reported by Han et al. (2017) through gross morphology observation was also supported by the greater granulation tissue formation and collagen synthesis in NS group compared to HP and the control. However, the differences in term of epithelialization, angiogenesis, and inflammatory cell infiltration at day 14 in NS group was not statistically significant compared to the control group [41]. These findings suggested that the wound-closure effect in NS is dependent on its tissue growth stimulation properties.

Improvement in the wound contraction rate can be at the expense of the tissue regeneration quality. In the study of excisional wounds by Kumandaş et al., NS and zinc–silver cream were reported to have slower wound closure compared to the control [42]. However, when the healed tissue was analysed, NS oil cream was found to induce the greatest epithelialization while maintaining lowest level of vascularization and inflammation [42]. 

As for diabetic wounds, Sari et al. described the positive effect of NS in the wound bed, where it portrayed less inflammation with low presence of polymorphonuclear neutrophil infiltration in NS-treated group compared to control group. Furthermore, more fibroblasts infiltration and epithelialization were reported at day 7 [43]. This was similar in the Nourbar et al. study, whereby epidermal thickness, collagen fibres, and fibroblast infiltration were the highest in NS group [45]. However, Yusmin et al. revealed TQ-treated group had more inflammation and low infiltration of fibroblasts at post-wounding day 14, which indicates the beginning timeline of fibroblast recruitment, thus a little behind than the control which was Vaseline [44].

Nevertheless, microscopically, it was proven that NS or TQ was better than the gold standard for burn treatment and showed better healing in excisional wounds. However, the effect in diabetic wounds was inconsistent possibly due to the involvement of multi-factorials including the lack formation of blood vessels (angiogenesis). This scenario at last would slow down the wound healing process that could trigger the presence of secondary infections due to the impairment of peripheral circulation, neuropathy, immunocompromised condition, and lead to a lesser extent hygienic concern due to obesity, older age, and inability to move [43,44,45].

#### 3.3.3. Biochemical Analysis

The state of oxidative stress in a tissue can be determined by measuring the total antioxidant state (TAS) and total oxidant stress (TOS) values. Oxidative stress markers include malondialdehyde (MDA), while antioxidant markers include glutathione (GSH), CAT, GPx, and SOD [59]. A decrease in TAS levels and increase in TOS levels denotes oxidative stress [36]. Prolong oxidative stress in the context of wound healing can attenuate the wound healing machinery. Only three studies reported the biochemical outcome of NS treatment in their wound healing model [41,42,46].

Burn injury has been associated with an increase in oxidative stress in the wound bed [60]. Consequently, the use of antioxidants significantly decreases burn mortality [61]. Using burn and excisional injury in a rat model, NS and TQ treatments lead to significantly high TAS and low TOS levels proving good antioxidant properties. MDA level significantly decreased while GSH, CAT, GPx, and SOD level significantly increased. In addition, NS and TQ outnumbered SS, HP, and zinc–silver in reducing oxidative stress [41,42,46]. Taken together, NS and TQ is a potent antioxidant that could accelerate wound healing rate.

## 4. Discussions

The systematic literature search revealed the current state of evidence regarding the effects of *Nigella sativa* (NS) and its bioactive compound, thymoquinone (TQ) on wound healing. Both NS and TQ affect wound healing differently depending on the type of wounds. NS’s effect was negligible in one diabetic wound study [43] and resulted in slower healing in another excisional wound study [42]. In terms of TQ, its wound healing enhancement effect was reported in one burn wound study [48], while another study reported TQ slowing down the wound healing in the later stage of diabetic ulcers [44].

Regarding description of the wound depth, Yaman et al. reported that their second-degree skin burns created a full-thickness wound. However, definition of second-degree burn is specific to a burn that caused a deep partial-thickness wound on the skin [61,62,63]. Moreover, four studies [14,41,42,44] did not specify any details about the depth of wound. As a result, drawing a conclusion on the effect of NS and TQ towards different wound thicknesses is impractical.

Therapeutic use of NS as a multipurpose “drug” has been widespread since its origin in the ancient Middle East through topical or oral therapy. TQ is the active compound of NS and is responsible for its anti-inflammatory, antioxidant, antibacterial, and anticancer properties [7]. The new challenge in wound healing therapy currently involves the metabolic impairment as a result of sedentary lifestyle and the emergence of resistant strains of bacteria [64]. The uses of NS for wound healing intervention need to be extensively studied and rediscovered.

Yusmin et al. and Sari et al. reported TQ/NS increases wound healing in the inflammatory phase of diabetic ulcers [43,44]. In diabetic condition, hyperglycaemia causes a dysfunctional inflammatory response due to the presence of neutrophil inflow that releases cytotoxic enzymes and inflammatory mediators. This mechanism will lead to discrepancy in reactive oxygen species (ROS) and subsequent oxidative stress [45]. Thus, TQ was found to accelerate wound healing in this phase, attributed mainly to its anti-inflammatory properties. It significantly reduced inflammation and improved re-epithelialization in a diabetic ulcer model. Furthermore, its antimicrobial properties minimized the risk of infection in early phases and could accelerate the wound healing process [65]. The antimicrobial properties of NS can also be attributed to *p*-cymene and carvacrol.

Although TQ accelerates wound healing in early phases, Yusmin et al. described wound healing at days 7 and 14 to be slowing down [44]. A hyperglycaemic condition in diabetes potentially effects the normal endothelial cell function and subsequently disrupts angiogenesis [44]. The wound becomes chronically inflamed and cannot progress to the granulation phase due to an imbalance between ROS and oxidative stress leading to lipid peroxidation and further disruption of fibroblast and endothelial cell function [62]. TQ was found to decelerate wound healing in this phase, mainly due to its anticancer properties which inhibit angiogenesis. Disruption of angiogenesis thought to be selective, affects only cancer cells but not normal cells. This predicted selective effect of TQ was based on a study showing toxicity in normal cells at the minimal level [66].

For three other type of wounds in a normal metabolic condition, all seven studies reported NS/TQ accelerated the wound healing process in all phases. NS improves wound healing by decreasing the total and absolute white blood cells count and limiting tissue damage and bacterial spread [15]. It is known that free radicals hinder the healing process. NS oil was found to reduce tissue malondialdehyde and protein carbonyl levels while preventing inhibition of superoxide dismutase, glutathione peroxidase, and catalase enzymes, thus accelerating wound healing [42]. Furthermore, fatty acid component of NS such as oleic and linoleic acid maintain the water barrier and promote wound healing by selectively transferring in and out of the wound during the healing process [67]. Fatty acid activates neutrophil phagocytosis and releases cytokine and growth factors, leading to the improvement of wound healing [68,69].

A burn injury is associated with increased oxidative stress and morbidity [60,70]. Antioxidant property is one of the important factors contributing to an increase in wound healing rate [36]. A clinical study by Yan et al. on burn injury revealed that antioxidants reduce morbidity on burn injury [71]. In two studies on the level of antioxidants during skin injury, both TQ and NS demonstrated increase in TAS and decrease in TOS levels, showing high antioxidant property. Hosseinzadeh et al. reported that TQ and NS inhibit the lipid peroxidation process in cerebral ischemia and reperfusion injury in rats [72]. Furthermore, Vorauer-Uhl et al. reported that application of SOD topically decreases the necrotic area and increases epithelialization rate in burn wounds [73].

However, when compared, honey and *Aloe vera* (AV) are superior to NS in terms of wound healing effect [40,43]. Honey is a low-pH substance and possesses anti-inflammatory, antioxidant, and antimicrobial properties that contribute to wound healing [40]. While in AV, anthraquinone is believed to be responsible for an anti-inflammatory effect, which accelerates wound healing [43]. The underlying mechanisms are revealed due to the inhibition of the cyclooxygenase pathway and the decrease in production of prostaglandin E2 from arachidonic acid [74]. Chithra et al. revealed that AV could increase the amount of collagen in granulation tissue and enhance the development of glycosaminoglycans and proteoglycans in the wound area [75].

From the literature search, a scarce amount of literature was available. At the current state, it is difficult to draw any conclusion on the effect of NS and TQ on wound healing. Hence, more studies need to be done in the future.

## 5. Conclusions

This review suggests that NS and TQ could have a significant role in accelerating wound healing, depending on the metabolic conditions.

NS’s positive effect in wound healing is supported by seven studies with only one study reporting its negligible effect. In terms of TQ, one study reported its wound healing acceleration effect, while another study reported its deceleration of wound healing, though, only in the proliferation stage, most probably due to its antiangiogenic property. All studies have variation in terms of country of origin. NS accelerates wound healing due to the anti-inflammatory, antioxidant, and antibacterial properties of its active constituent; TQ. Further study needs to be performed to clarify the mechanisms involved in wound healing from various sources of NS and TQ.

## Figures and Tables

**Table 1:** Summary of *Nigella sativa* (NS) data.

References	Experimental Model	Form of NS	Methods	Results	Conclusion
Kumandaş et al., 2019 [42]	Excision wound in male Wistar-albino rats	NS oil cream	Treatment groups: NS oil cream group (NS). Zinc–silver cream group (ZnAg). Saline group (Control). Parameters: Gross morphology analysis for time to healing. Biochemical analysis at day 0, 3, 7, and 14 for measurement of malondialdehyde (MDA), catalase (CAT), and superoxide dismutase (SOD). Histology assessment at day 7 and 14 for inflammation, vascularization, and changes in the epithelium.	Outcomes: Time to healing from fastest to slowest were Control, ZnAg, and NSO. MDA and CAT levels from highest to lowest were Control, NS, and ZnAg; while NO levels from highest to lowest were ZnAg, NS, and Control. Epithelialization from highest to lowest were NSO, Control, and ZnAg; whereas inflammation and vascularization from highest to lowest were ZnAg, Control, and NSO.	NS oil caused better epithelialization and granulation tissue while reducing vascularization and inflammation during wound healing.
Nourbar et al., 2019 [45]	Chronic delayed wound in streptozotocin-induced diabetic rats	NS extract	Treatment groups: Nondiabetic untreated group (NU). Nondiabetic treated with 1% phenytoin (NP). Eucerin treated sham (NE). Diabetic untreated (DU). Diabetic treated with 1% phenytoin (DP). Diabetic treated with 20% NS extract (DNS20). Diabetic treated with 40% NS extract (DNS40). Parameters: Gross morphology analysis for time to healing. Histology assessment at complete healing for epidermal thickness, collagen fibres, and fibroblast infiltration.	Outcome: Time to healing from fastest to slowest were DNS40, DNS20, NP, NU, DP, NE, and DU. Epidermal thickness, collagen fibres, and fibroblast infiltration were highest in DNS40.	NS extract could accelerate wound healing in streptozotocin-induced diabetic rats.
Elgohary et al., 2018 [48]	Chemical burn using concentrated HCl (38%) in albino rabbits	NS oil	Treatment groups: Pulsed ultrasound group (PU). Continuous ultrasound group (CU). Topical NS oil group (NS). Pulsed phonophoresis using NS oil group (PPNS). Continuous phonophoresis using NS oil group (CPNS). Moist exposed burn ointment group (Positive control). Normal saline group (Negative control). Parameters: Gross morphology analysis at day 0, 5, 10, 15, and 20 for necrotic tissue area.	Outcomes: After 5, 10, 15, and 20 days, wound area from smallest to largest were PPNS, Positive control, NS, CPNS, PU, CU, and Negative control group.	Pulsed phonophoresis using NS oil can be used as an adjunct treatment with limited side effects to promote wound contraction and inhibit inflammation; thus, accelerating wound healing.
Javadi et al., 2018 [40]	Excision wound in male Wistar-albino rats	Cold pressed NS seed oil	Treatment groups: Lanolin group (Negative control). Honey group. NS group. NS and honey group (Mix). Phenytoin group (Positive control). Parameters: Gross morphology analysis at day 0, 5, 10, 15, and 20 for necrotic tissue area.	Outcomes: Necrotic tissue areas at 5, 10, 15, and 20 days post-wounding from largest to smallest were Lanolin, NS, Honey, Phenytoin, and Mix groups. The mix group was significantly lower than the other groups in all post-wounding days.	NS seed oil can accelerate wound healing and the effect is greater in combination with honey.
Sari et al., 2018 [43]	Chronic delayed wound in alloxan-induced diabetic male Wistar rats	NS oil gel	Treatment groups: 100 µL of NS oil gel group (NS). 100 µL of *Aloe vera* oil gel group (AV). Untreated group (Control). Parameters: Gross morphology observation daily for 7 days for wound area. Histology assessment at day 7 for inflammation, re-epithelialization, and fibroblast infiltration.	Outcomes: At day 6 and 7, AV group have significantly smaller wound size compared to NS and control group. No difference in wound size was observed between the three groups at day 1 to 5. On day 7, there was less intense inflammation, more fibroblasts infiltration, and more complete re-epithelialization in AV group compared to NS group and control group.	NS oil gel has negligible effect on wound healing in diabetic rats.
Han et al., 2017 [41]	Full thickness wound in female Wistar-albino rats	NS oil cream	Treatment groups: Placebo cream group (Control). 50% NS oil cream group (NS). 50% *Hypericum perforatum* oil cream group (HP). Parameters: Gross morphology analysis at day 7 and 14 for wound area and wound contraction. Histology assessment at day 7 and 14 for inflammation, angiogenesis, re-epithelialization, thickness of granulation tissue, and accumulation of collagen. Biochemical analysis at day 7 and 14 for measurement of MDA, GSH, CAT, GPx, and SOD.	Outcomes: On day 7 and 14, NS group demonstrated the smallest wound area and the highest wound contraction to HP and control group. Lesser degree of inflammation was found in NS group and HP group compared to control group while greater angiogenesis, re-epithelialization, granulation tissue and collagen accumulation were found in HP group compared to NS and control group. On day 7 and 14, MDA levels were significantly lower in NS group compared to control group while GSH, CAT, GPx, and SOD levels were significantly higher in NS group compared to control group.	NS exerts a wound healing effect through its antioxidant property, in contrast to HP that enhances wound healing via epithelialization and granulation-encouraging effects.
Shahani et al., 2013 [14]	Cutaneous wound in Wistar rabbits	NS extract oil	Treatment groups: NS extract oil group (NS). 1% pyodine group (Control). Parameters: Histology assessment at day 5, 9, and 14 for inflammation, angiogenesis, granulation tissue formation, fibroblast proliferation, and collagen synthesis.	Outcomes: On day 5, 9, and 14, there were significantly greater granulation tissue formation, angiogenesis, fibroblasts proliferation, and collagen synthesis in NS extract oil group compared to control group.	NS extract oil induces angiogenesis, fibroblasts proliferation, and collagen synthesis during wound healing in rabbit.
Yaman et al., 2010 [47]	Burn wound model in male Wistar-albino rats	NS oil	Treatment groups: NS group. SS group. Cold cream group (Control). Parameters: Histology assessment at day 0, 4, 9, and 14 for degree of inflammation and thickness of granulation tissue.	Outcomes: Inflammation was less apparent in NS and SS groups compared to control group from day 0 to day 14 while thickness of granulation tissue was significantly higher in NS compared to SS group and control group at day 9 and 14.	NS was found to accelerate healing process via its antimicrobial, antioxidant, anti-inflammatory, and immunomodulatory effects.

**Table 2:** Summary of thymoquinone (TQ) data.

References	Experimental Model	Form of NS	Methods	Results	Conclusion
Yusmin and Ahmad 2017 [44]	Chronic delayed wound in alloxan-induced diabetic rats	TQ in petroleum jelly	Treatment groups: TQ group (TQ). Vaseline group (Control). Parameters: Gross morphology analysis at day 3, 7, and 14 for wound contraction. Histology assessment at day 14 for inflammation, angiogenesis, granulation tissue, and deposition of collagen.	Outcomes: On day 3, wound contractions in TQ group was significantly greater than control group but significantly lower than control group at day 7 and 14. On day 14, TQ group has significantly lower inflammation and greater granulation tissue compared to the control group. Angiogenesis and collagen depositions were similar for both groups.	TQ accelerated wound healing during the inflammatory phase but decelerated wound healing during the granulation phase in diabetic rats.
Selçuk et al., 2013 [46]	Deep second degree burn in Sprague–Dawley rats	Topical and intraperitoneal delivery of TQ	Treatment groups: Untreated group (Control). SS group. 2 mg/kg/day intraperitoneal TQ group (Systemic). 0.5% topical TQ group (Topical). Systemic and topical TQ group (Combination). Parameters: Gross morphology for wound size at day 5, 7, 10, 15, 18, and 21. Histology assessment at day 21 for degree of inflammation, vascularization, re-epithelialization, and thickness of granulation tissue. Biochemical analysis of total antioxidant state (TAS) and total oxidative stress (TOS).	Outcomes: At end of day 21, none of the wounds closed completely. The order of wound size from largest to smallest were Control, Systemic, SS, Topical, and Combination. The granulation tissue formation and vascularization were significantly lower in control group compared with other groups while the inflammatory cell response and epithelialization was highest in control group and SS group and lower in TQ group. TAS levels in TQ group were significantly higher than control group while TOS levels in TQ group were significantly lower than control and SS group.	TQ appears to accelerate the rate of wound closure both in topical and systemic administrations, and this effect is stronger for the topical administration.
